# Development of a therapist-supervised flexibility and resistance exercise intervention for adults aged 50 years and over with a distal radius fracture in the Wrist Injury Strengthening Exercise (WISE) trial

**DOI:** 10.1302/2633-1462.710.BJO-2026-0101.R1

**Published:** 2026-10-20

**Authors:** Warren Sheehan, Sarah E. Lamb, Colin P. Forde, Cynthia Srikesavan, Phillipa J. A. Nicolson, Elizabeth Tutton, Jenny Gould, Richard Grant, Juul Achten, Duncan Appelbe, Matthew L. Costa, David J. Keene

**Affiliations:** 1 Oxford University Hospitals NHS Foundation Trust, Oxford, UK; 2 Public Health and Sport Sciences, University of Exeter, Exeter, UK; 3 Kadoorie Institute of Trauma, Emergency and Critical Care, Nuffield Department of Orthopaedics, Rheumatology and Musculoskeletal Sciences, University of Oxford, Oxford, UK; 4 Botnar Institute for Musculoskeletal Sciences, Nuffield Department of Orthopaedics, Rheumatology and Musculoskeletal Sciences, University of Oxford, Oxford, UK

**Keywords:** Rehabilitation, Exercise, Advice, Therapy, Distal radius fracture, Distal radius fractures, resistance exercises, Wrist Injury, randomized controlled trial, physiotherapists, supervised rehabilitation, exercise programmes, wrist, immobilization, upper-limb

## Abstract

**Aims:**

The definitive Wrist Injury Strengthening Exercise (WISE) trial is a multicentre superiority randomized controlled trial (RCT) evaluating usual care advice plus a therapist-supervised flexibility and resistance exercise intervention versus usual care advice alone for adults aged ≥ 50 years with a distal radius fracture (DRF). This paper describes the development and delivery of the therapist-supervised exercise intervention evaluated in the definitive WISE RCT.

**Methods:**

The intervention was developed iteratively in accordance with Medical Research Council (MRC) guidance for complex interventions. UK DRF clinical guidelines, published rehabilitation RCTs, UK NHS patient information leaflets, common post-injury impairments, and exercise and behavioural theory were reviewed to inform candidate intervention components. Feedback from UK therapists and patient and public involvement partners then informed the design of the initial intervention. Two methods of intervention delivery were then evaluated in a multicentre feasibility RCT and embedded qualitative study involving 117 participants aged ≥ 50 years with a distal radius fracture.

**Results:**

Review of guidelines and previous research highlighted uncertainty regarding the optimal rehabilitation after DRF and limited reporting of progressive exercise interventions. NHS patient information leaflets largely focused on flexibility exercises and self-management advice. Therapist and patient feedback informed the intervention content, progression, and participant materials. Findings from the feasibility RCT and embedded qualitative study supported a therapist-supervised delivery model with three sessions, rather than a single-session independent exercise approach. The final intervention comprised usual care advice plus up to three one-to-one sessions with a physiotherapist or occupational therapist over 12 weeks. Participants also received a workbook containing self-management advice, progressive flexibility and resistance exercises with resistance bands and therapeutic putty, and behaviour change strategies to support exercise adherence.

**Conclusion:**

Using an iterative MRC-informed process, a therapist-supervised rehabilitation intervention was developed and refined prior to definitive evaluation. Transparent reporting of intervention development and content should support interpretation, replication, and implementation.

Cite this article: *Bone Jt Open* 2026;7(10):1352–1364.

## Introduction

Distal radius fractures (DRFs) account for 18% of adult fractures seen in UK hospitals.^1^ The average age of injury is 56 years due to the high proportion of older females affected.^1^ Initial treatment is non-surgical with or without manipulation, or surgical fixation. After both approaches, the wrist is temporarily immobilized in a cast or splint. Following removal of this initial immobilization, many patients experience pain, stiffness, and muscle weakness which can make performing everyday tasks difficult.^2^ Some symptoms may even persist for up to one year.^3^ Older female patients also have a 48% increased risk of a clinically important long-term functional decline, as measured by the ability to perform activities like food preparation, heavy housework, and shopping.^4^

Although DRFs are extremely common among older adults, there is insufficient high-quality randomized controlled trial (RCT) evidence to inform rehabilitation provision after the initial immobilization period.^5^ Recent prediction modelling using Scottish national data suggests that the annual number of DRFs in older adults will nearly double by 2040, highlighting the importance of establishing evidence-based and scalable rehabilitation strategies for this population.^6^ Previously, the Strengthening And stretching for Rheumatoid Arthritis (SARAH) RCT showed that adding an upper limb flexibility and hand strengthening exercise intervention to usual care was clinically and cost-effective compared to usual care alone for people with rheumatoid arthritis who had hand pain and dysfunction.^7^ Considering this finding, several principles of the SARAH programme were drawn on to produce a new intervention for adults aged 50 years and over with a DRF. Two different ways of delivering this new intervention were then tested in the Wrist Injury Strengthening Exercise (WISE) feasibility RCT: by a physiotherapist or occupational therapist (hereafter referred to as ‘therapists’) in a single session, or over three sessions.^8^

Based on that feasibility trial’s findings, the definitive WISE RCT was designed. This pragmatic, parallel-group, superiority RCT compared usual care advice plus a therapist-supervised flexibility and resistance exercise intervention (delivered over three sessions) versus usual care advice alone for adults aged ≥ 50 years with a DRF. Full trial details are in the published protocol.^9^ In this paper, the development and delivery of the therapist-supervised exercise intervention evaluated in the definitive WISE RCT is described. Complex rehabilitation interventions are frequently insufficiently described in trial reports, limiting replication, implementation in clinical practice, and interpretation of trial findings irrespective of whether effectiveness results are positive or null. Current Medical Research Council (MRC) and National Institute for Health and Care Research (NIHR)-funded guidance therefore recommends transparent reporting of intervention development processes and intervention content, alongside definitive evaluations of complex interventions.^10^ The aim of this paper was therefore to describe the development, refinement, and delivery of the therapist-supervised WISE intervention evaluated in the definitive RCT.

## Methods

The finalized WISE therapist-supervised exercise intervention was developed iteratively and in stages (see Figure 1). This process was in accordance with the MRC’s 2008 guidance on developing complex interventions, subsequently updated in 2021.^11^ First, UK DRF clinical guidelines, exercise programmes in published rehabilitation RCTs, UK NHS patient information leaflets, common post-injury impairments, and relevant scientific theory were examined. Considering this information, candidate intervention advice and exercise components were identified. Feedback on these were sought from experienced NHS therapists via electronic survey. Drawing on findings from the preceding stages, the intervention was designed, aiming for deliverability and scalability across the UK NHS, and two ways of delivering it were tested in a feasibility RCT and embedded qualitative study. Based on the feasibility study’s findings, the therapist-supervised exercise intervention was refined and evaluated in the definitive WISE RCT. Patient and public involvement (PPI) partners contributed throughout the intervention development. The intervention development process is described in detail below.

**Fig. 1:**
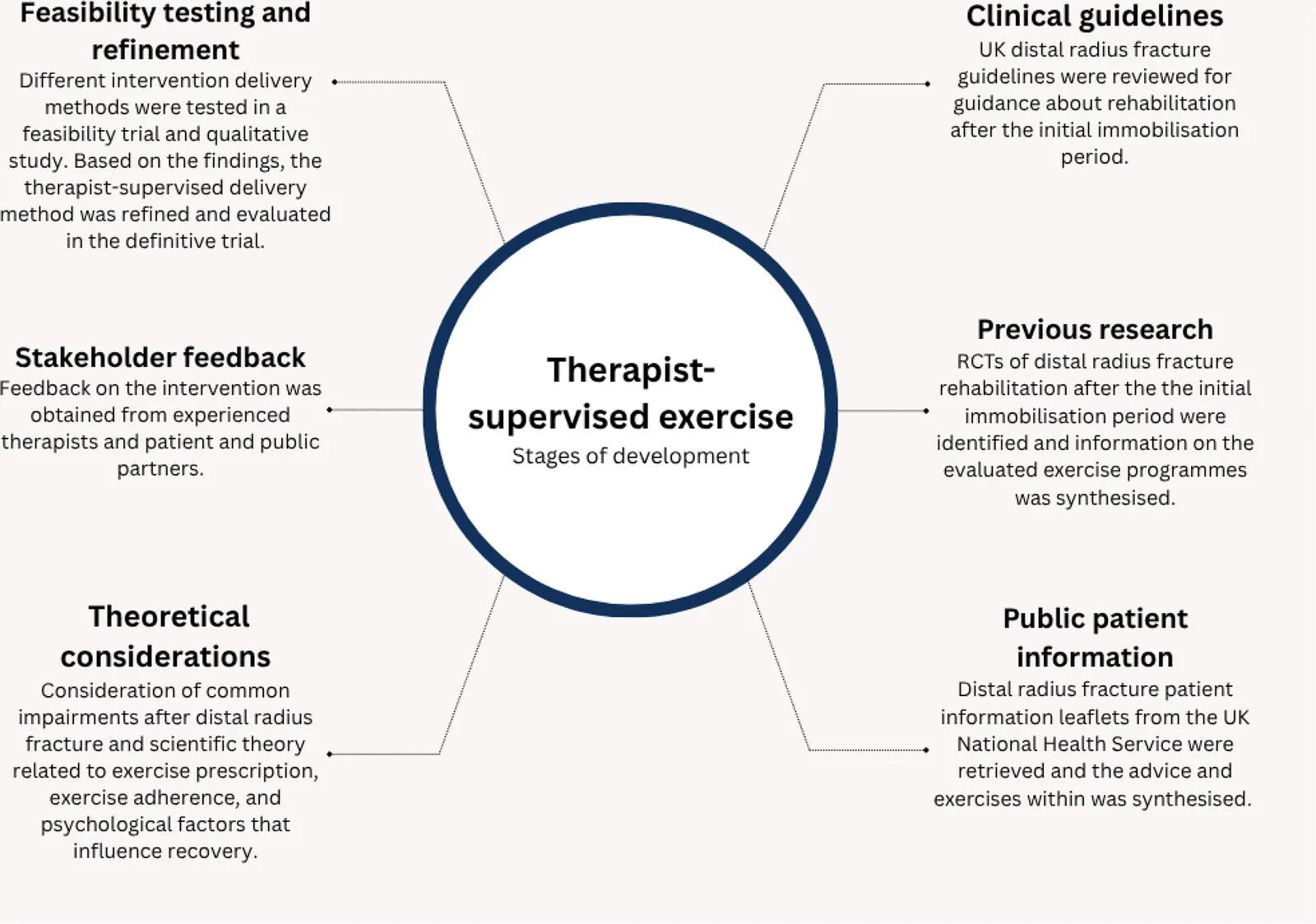
Overview of the intervention development process.

### Intervention development

**Stage 1a:** Clinical guidelines: To inform the intervention development,^12-14^ clinical guidelines from the UK on DRF care were identified via searches of the National Institute for Health and Care Excellence (NICE) and British Orthopaedic Association (BOA) and associated specialist societies resources.^12-14^

**Stage 1b:** Previous published research: There is insufficient high-quality evidence to support routine referral for therapy or the use of specific types of exercises for older adults after DRF (see the definitive WISE trial protocol^9^ for a summary of the existing evidence and its limitations). To inform the exercise component of the intervention, systematic reviews were screened^5,15^ and PubMed, PEDro, and the Cochrane Central Register of Controlled Trials databases were searched in 2020 for DRF RCTs that evaluated exercise after initial immobilization. Data was extracted on the exercises and prescription parameters, exercise equipment, and exercise progressions.

**Stage 1c:** UK NHS patient information: To help understand what advice and exercises are given to adults after DRF in the UK NHS, Google (USA) was used in April 2020 to search for UK NHS DRF patient information leaflets.

**Stage 1d:** Theoretical considerations: physical impairments and underpinning exercise and behavioural science.^16^ Available evidence regarding common physical impairments and exercise and behavioural theory was considered to inform intervention design.

**Stage 2a:** Stakeholder feedback: Survey of UK therapists: The limited high-quality evidence to inform rehabilitation meant obtaining feedback from experienced therapists was vital to optimize the intervention’s content and to increase the chances that it would be acceptable to therapists and deliverable in clinical practice. An in-person intervention development workshop with therapists was planned, but due to the COVID-19 pandemic, views were obtained via an online survey instead. In June 2020, a survey link was emailed to therapists working in the UK NHS who had expressed an interest in supporting the intervention’s development. The survey asked participants to rank possible intervention: 1) advice topics; 2) flexibility exercises; 3) resistance exercises; and 4) exercise prescription parameters. Therapists could also use free text boxes to suggest additional advice topics and exercises to include.

Patient and public involvement: During development of the study, the WISE study team met with members of the UK Musculoskeletal Trauma PPI group to hear their views on the intervention and study design.

**Stage 2b:** Designing the intervention: Three physiotherapists on the research team (DJK, PN, CS) discussed the findings from the preceding stages then developed an initial prototype intervention. Feedback on this prototype was obtained from the wider study team, clinicians, and PPI partners. The senior author (DJK) made the final decision about the intervention content and materials.

**Stage 3:** Feasibility RCT and embedded qualitative study: The feasibility RCT recruited 117 participants aged ≥ 50 years with DRFs from six NHS hospitals. Different methods of intervention delivery were assessed by randomly allocating participants to: 1) usual care advice; 2) usual care advice plus independent exercise (delivered by a therapist in one session); or 3) usual care plus supervised exercise (delivered by a therapist in up to three sessions). Semi-structured interviews were undertaken with purposively sampled participants and treating therapists involved in the feasibility trial to explore intervention acceptability, barriers and facilitators to exercise participation, and views regarding intervention delivery. Interviews were conducted by an experienced qualitative researcher (ET) using topic guides informed by the study aims. Data were analyzed thematically using an inductive approach. Detailed qualitative findings will be reported separately. However, key findings relevant to intervention refinement are summarized here.

### Intervention reporting

The therapist-supervised exercise intervention is reported below, following the Template for Intervention Description and Replication (TIDier)^17^ and Consensus on Exercise Reporting Template (CERT).^18^

## Results

### Intervention development

**Stage 1a:** Clinical guidelines: At the time of intervention development, two UK DRF guidelines included information about rehabilitation. The BOA and British Society for Surgery of the Hand guidelines^12^ found no robust evidence to guide rehabilitation provision after initial fracture treatment. In the absence of evidence, the guidance recommends that patients are given information, which should cover common complications and how to manage them, to enable patients to self-manage their recovery. It was indicated that referring patients for rehabilitation provided by a therapist should be reserved for patients with persistent or disproportionate pain, stiffness, and disability. The only guidance about rehabilitation in the BOA Standards for DRFs is that patients should receive information about their recovery and returning to functional activities, and be able to re-access fracture services if required.^13^ Currently, there is no guidance on rehabilitation after initial DRF treatment in the NICE guidelines on the assessment and management of non-complex fractures.^14^

**Stage 1b:** Previous published research: In all, 17 RCTs were identified. Details of included exercises, how they were to be performed, initial dosage, and progressions were generally poorly reported. When reported, there was variability in the number, type, and dose of exercises prescribed. Most exercise programmes focused on improving forearm, wrist, and finger range of motion (ROM). Only 8/17 RCTs reported using resistance exercises in the injured arm and none targeted proximal upper limb muscle strength. Only 8/17 RCTs reported using resistance exercises in the injured arm and none targeted proximal upper limb muscle strength, though occasionally functional task practice was used.

**Stage 1c:** UK NHS patient information: Leaflets from 23 UK NHS trusts were retrieved; 14 leaflets contained advice on recovery and eight focused on exercises to be done in the cast. Exercises for the post-immobilization period focused on improving finger and wrist ROM. Only one patient information leaflet included resistance exercises (grip and pinch strengthening exercises), and none had an exercise progression plan.

**Stage 1d:** Theoretical considerations: Flexibility: Hand function partly depends on being able to move the upper limb joints freely so that they can place the hand precisely in space.^19^ Indeed, artificially constraining movement at the finger, wrist, forearm, and elbow joints individually impairs hand function.^19^ The intervention therefore targeted the flexibility of these joints which can be stiff to varying degrees after DRF.^20,21^ The exception was elbow flexibility, which the study team decided not to target because elbow stiffness is comparatively rare, in the study team’s clinical experience, and considering the need to minimize the number of prescribed exercises to aid intervention delivery and participants’ exercise adherence.

Muscle strength: Functional limitations after DRF relate to difficulty with tasks involving heavy gripping, lifting, and pushing. For example, opening jars and doors, gardening, and playing golf.^22^ These experiences of difficulty returning to more demanding activities were also highlighted by our PPI partners. Furthermore, research has shown that grip strength deficits compared to the uninjured side can last for up to two years after injury,^23^ and grip strength is more closely associated with upper limb pain and function than wrist ROM.^21,24^ Proximal muscle weakness from disuse is also likely and could contribute to disability. Despite this, as outlined above, there is limited evidence about the effectiveness of resistance training in this patient group and its use in NHS practice appears limited.

In resistance training, the two foremost principles are ‘progressive overload’ and ‘specificity’.^16^ Progressive overload states that a gradually more challenging training stimulus is necessary to stimulate ongoing physiological adaptation.^16^ This can be achieved by manipulating training variables like exercise intensity, volume, and duration. Therefore, within a structured exercise programme for people with different physical capabilities and symptoms, some training variables must be modifiable to achieve a sufficient training stimulus. ‘Specificity’ states that the adaptations from training directly relate to the type of training done.^16^ This provided a rationale for incorporating resistance exercises for the whole upper limb that replicate functional activities known to be challenging after DRF (e.g., gripping, pulling, and pushing).

Strategies to promote adherence to prescribed exercise and psychological factors: To achieve a sufficient training stimulus for physiological adaptation, patient adherence to prescribed exercise is important. Despite this, adherence varies.^25^ Psychological factors, including pain catastrophising and pain self-efficacy, are also associated with disability after upper limb injury.^26^ However, exercise adherence, pain catastrophising, and self-efficacy are potentially modifiable, so strategies to address these were included in the intervention.

In the SARAH trial,^7^ participants allocated to the exercise intervention reported higher exercise adherence and self-efficacy than the usual care group. These effects were likely attributable, in part, to the advice provided and the simple evidence-based behaviour change strategies used to promote exercise adherence (including setting personalized goals, planning where and when to do exercises, and providing a diary to record exercise adherence) which were based on those in the NHS Health Trainer Handbook.^27^ Several key principles from the SARAH intervention^28^ and the AFTER trial intervention^29^ (physiotherapist-supervised rehabilitation for people with ankle fractures) were drawn on, but these were adapted for the WISE intervention target population, including the baseline prescription and progression of resistance exercises and the behavioural framing to promote adherence.

**Stage 2a:** Stakeholder feedback: Survey of UK therapists: Overall, 20 mainly senior therapists responded (NHS agenda for change band 6 = 1, band 7 = 12, band 8 = 7). In all, 17 respondents were working clinically and 15 mainly treated patients with hand conditions. The survey findings (summarized in the Supplementary Material) informed the design of the initial prototype intervention (stage 2b).

Patient and public involvement: PPI partners identified that supporting people to exercise after a fracture was important. They highlighted experiences of “lacking confidence” after injury, “fearing that exercises could be damaging” particularly as they are “often sore”, and that motivation could be a challenge when exercising independently. The quality of information was seen as crucial for success. They felt a website could be a valuable alternative to a paper workbook in providing this information.

Two PPI members on the study team and other members of the UK Musculoskeletal Trauma PPI group reviewed all participant-facing intervention materials. There was a focus on ensuring the format, images, and text were accessible. During the website’s development, three PPI partners took part in telephone discussions. They emphasized the importance of: 1) keeping it simple; 2) having concise and useful information; 3) including photographs to support text; 4) using plain background with lots of white space; 5) having easy navigation features, and 6) producing videos with people relatable in daily life.

**Stage 2b:** Designing the intervention: Prior to the feasibility RCT, the interventions were developed and the materials for their implementation produced. There was a focus on balancing deliverability and scalability across the UK NHS, ensuring that the exercise programme was sufficiently challenging and progressive, and using evidence-based strategies to support participant exercise adherence.

**Stage 3:** Feasibility RCT and embedded qualitative study: The therapist-supervised method of intervention delivery was taken through to the definitive RCT phase instead of independent exercise. Participant and staff feedback highlighted that the extra therapy sessions were valuable opportunities for collaborative problem-solving between participants and therapists e.g. how to manage pain during exercise. Participants considered therapy sessions useful to demonstrate the exercises, ask questions, and gain confidence. Reviewing goals with therapists also provided reassurance that they were progressing. Intervention acceptability was high among treating therapists. However, they preferred to support progression through the exercise programme over three sessions rather than one.

In the feasibility RCT, some participants also found performing wrist exercises that required starting in supination or pronation challenging. So, for the main RCT, an option to perform these specific exercises in neutral forearm rotation was added. There were also minor amendments to the paper workbook (which were mirrored on the web-version), but the content remained essentially the same. For example, some phrasing was changed and the layout of the exercise section was refined for clarity. New higher-quality exercise demonstration photos were used, and quick-response (QR) codes and uniform resource locators (URLs) were added to the paper workbook which were linked to new exercise demonstration videos. The new exercise demonstration photos and videos were done in collaboration with a production company, Oxford Medical Illustrations at Oxford University Hospitals NHS Foundation Trust. These replaced the versions used in the feasibility phase, which were produced in-house by the team due to COVID-19 pandemic restrictions.

### Description of the WISE trial interventions

Usual care advice (comparator): This is the usual self-management advice given by an orthopaedic surgeon, therapist, or nurse in fracture clinic to patients with a DRF after removal of their cast or splint (normally four to eight weeks after injury). Typically, advice covered returning to functional activities and exercises to improve hand and wrist flexibility. The clinician could give the participant a patient information leaflet if this was part of their usual practice.

Therapist-supervised exercise (intervention): An overview of this intervention according to the TIDieR criteria is presented in Table I.

**Table I.** Overview of therapist-supervised exercise intervention according to Template for Intervention Description and Replication (TIDieR) criteria.

TIDieR item	Therapist-supervised exercise
Why	The intervention aimed to improve wrist pain and function by targeting upper limb flexibility and muscle strength, participant adherence to prescribed exercise, and self-efficacy. Delivery of the intervention by a therapist over multiple sessions enabled tailoring of the intervention components to individual participant’s characteristics, circumstances, and progress.
What	
Materials: participants	Following recruitment or at the first therapy session, participants received a paper-based workbook which they could also access online if they wanted to. The workbook contained self-management advice (Table II); an exercise programme (Table II); and an exercise planner (where participants set goals and planned where and when to do exercise) and diary to record exercise performance (Supplementary Material). To perform their resistance exercises, participants also received resistance bands and therapeutic putty.
Materials: therapists	Therapists received a manual which concisely described how to deliver the intervention and complete trial documentation.
Procedures	At session 1, following their normal assessment, the therapist explained to the participant the following sections and how to use them: advice, exercises, and strategies to promote exercise adherence. At review sessions, the therapist re-assessed the participant as they normally would, provided personalized feedback on progress, and tailored the advice, exercises, and exercise adherence strategies accordingly.
Who provided	Fully qualified NHS physiotherapists or occupational therapists at recruiting centres related therapy departments. They did not need specific expertise or experience, but all completed training on the intervention before providing it.
How	Therapy sessions were one-to-one. Initial sessions were face-to-face or by video, and review sessions were face-to-face or by video or telephone.
Where	Face-to-face sessions were in outpatient departments. Participants chose where to conduct video and telephone sessions.
When and how much	Usual care advice was given in fracture clinics upon removal of the cast or splint. The first intervention session was 40 to 60 mins and on the same day as usual care advice or as soon as possible afterwards (within 3 weeks). Review sessions were up to 30 mins and aimed to occurr within 12 weeks of the first session at a schedule agreed by the therapist and participant.
Tailoring	At each session, therapists: could provide any additional personalized advice judged relevant. used an effort scale (0 to 10) to select which resistance band and putty colour participants used for resistance exercises prescribed optional finger stretching exercise if deemed necessary At review sessions, therapists: provided tailored feedback to participants on progress with the exercise programme and return to activities identified any barriers to exercise and helped the participant problem solve how to overcome these reviewed the participant’s goals and exercise plan and helped them modify these as needed
Modifications	In the feasibility phase, some participants found performing some wrist exercises challenging because these were in supination or pronation. For the main trial, an option to perform these exercises in neutral forearm rotation if required was added. Some clarification in relation to information about pain was also included based on feedback from participant interviews.
How well	
Planned: therapists	See quality assurance section.
Planned: participants	Participant-reported questionnaires at 3 and 6 months after randomization asked participants how many times in the preceding 2 weeks they did exercises for their injured wrist.
Actual: therapists and participants	Therapists’ fidelity to the intervention and participants self-reported frequency of performing exercises will be reported in the results paper.

Mins, minutes.

**Table II.** Therapist-supervised exercise programme.

Category	No.	Prescription parameters	Demonstration photos
**Flexibility: Wrist and forearm**			
PROM wrist flexion then extension, option 1 (forearm pronated)	1	3 × 30 s, 3 times/day	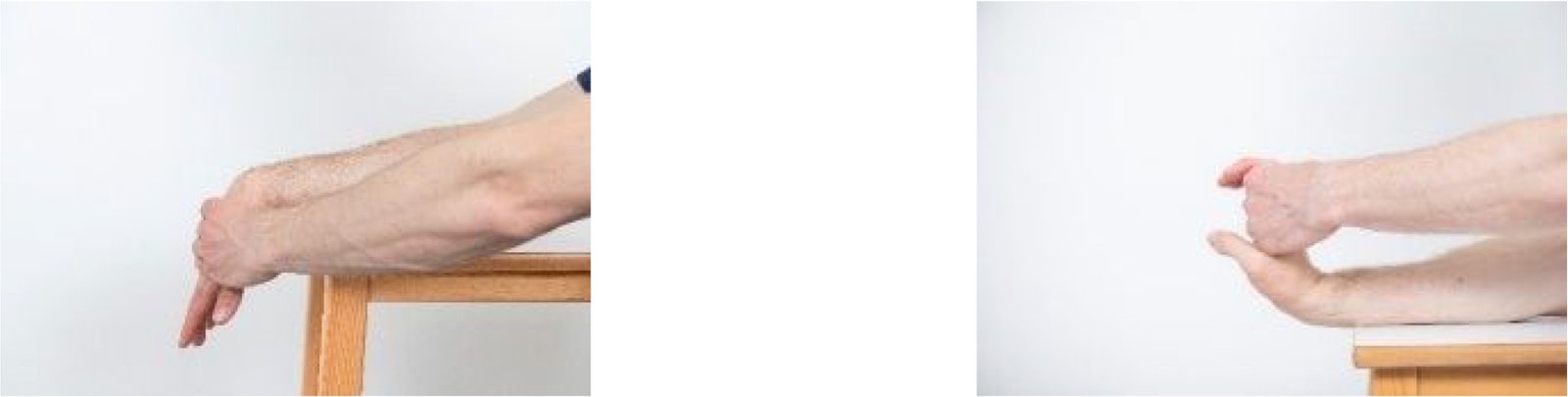
PROM wrist flexion then extension, option 2 (neutral forearm rotation)		3 × 30 s, 3 times/day	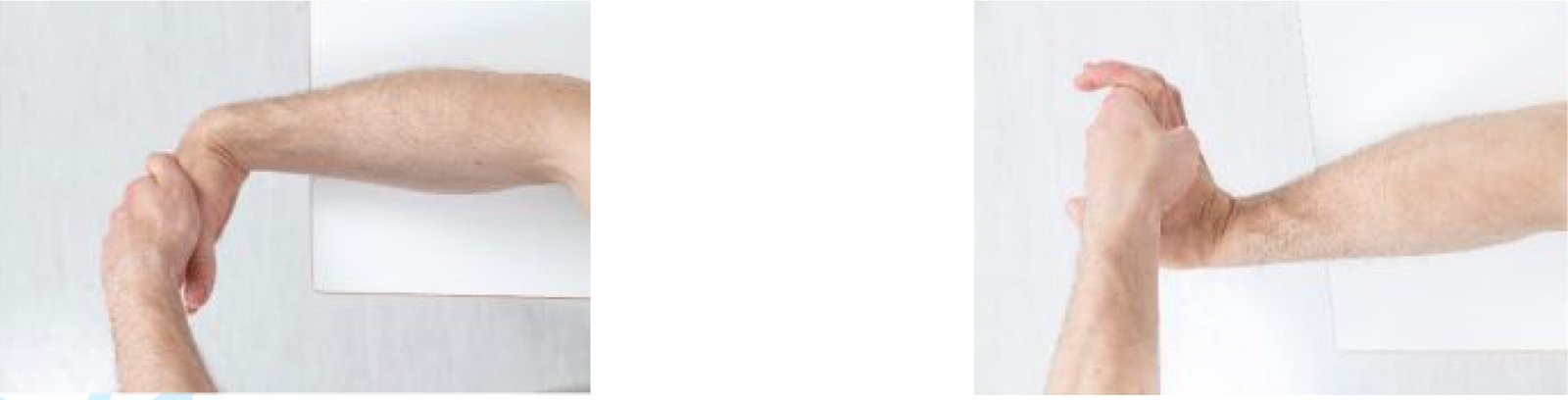
PROM ulnar then radial deviation, option 1 (forearm pronated)	2	3 × 30 s, 3 times/day	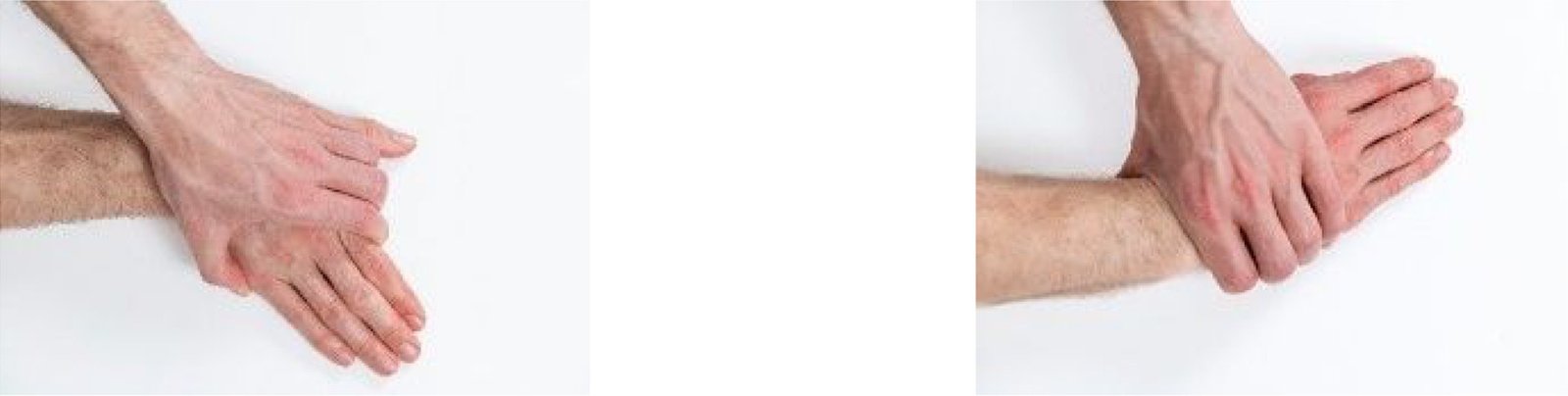
PROM ulnar then radial deviation, option 2 (neutral forearm rotation)		3 × 30 s, 3 times/day	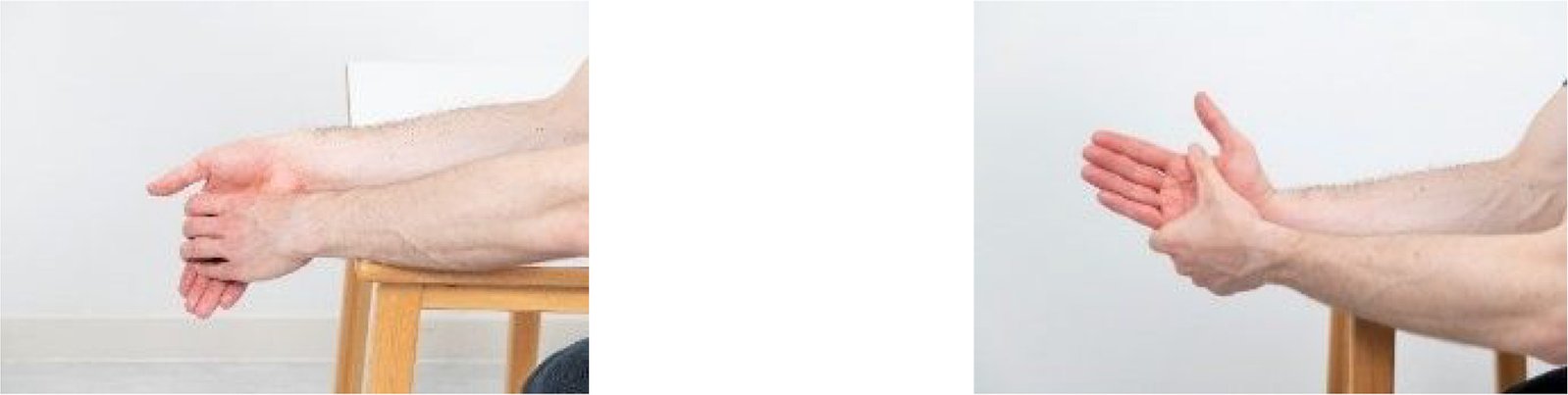
PROM forearm pronation then supination	3	3 × 30 s, 3 times/day	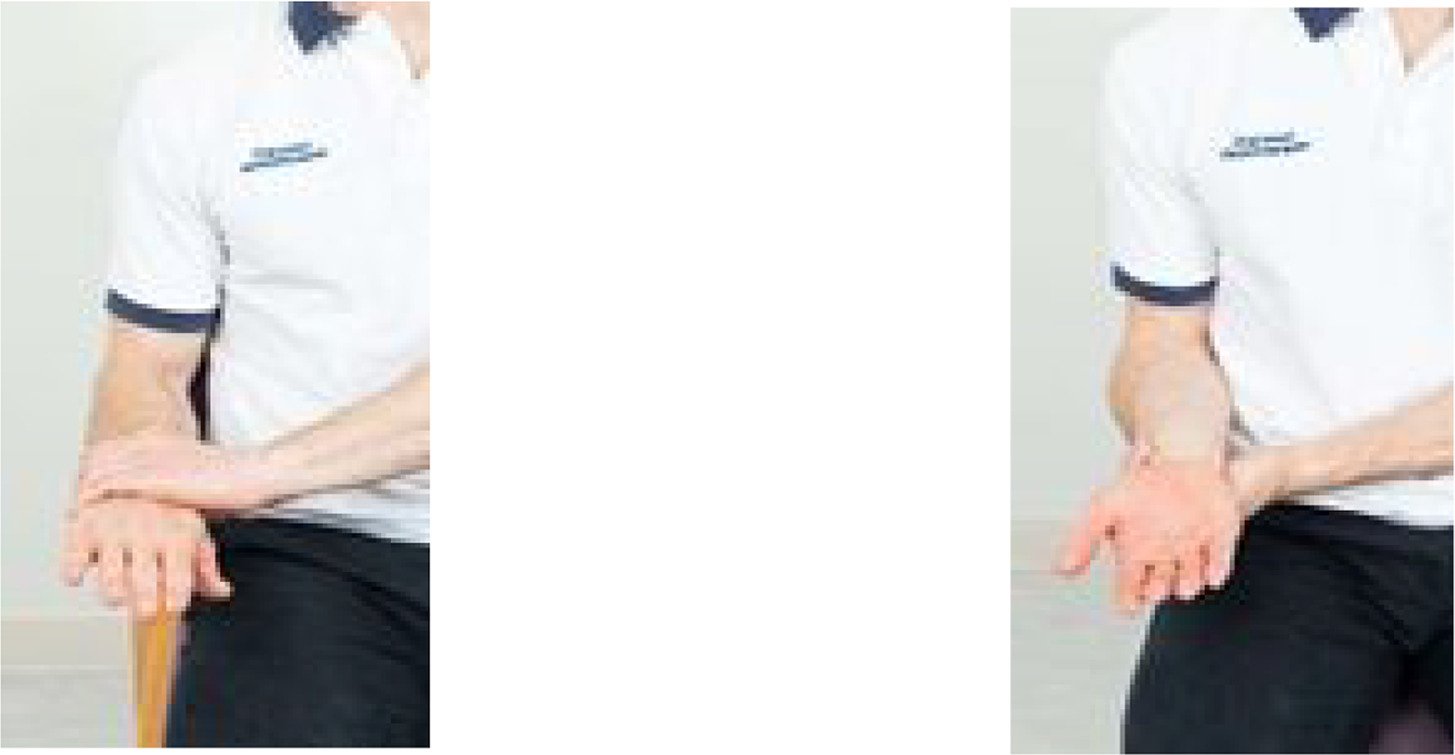
PROM wrist extension (prayer stretch)	4	3 × 30 s, 3 times/day	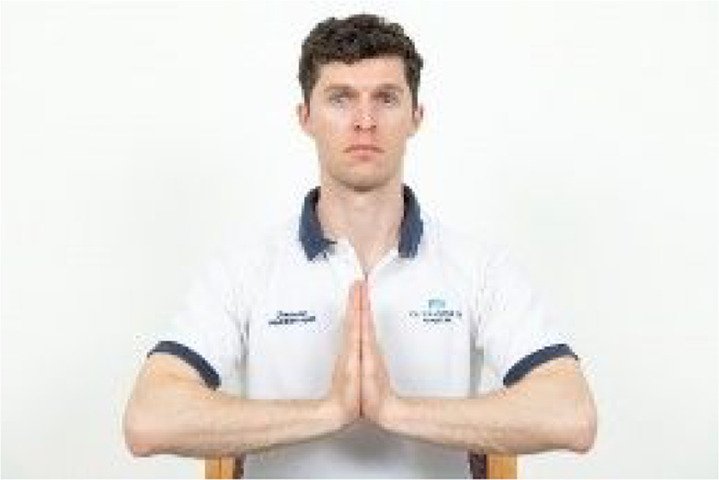
**Optional flexibility: fingers**			
AROM MCP extension then flexion (tabletop)	1	1 set x 10 reps, 3 times/day	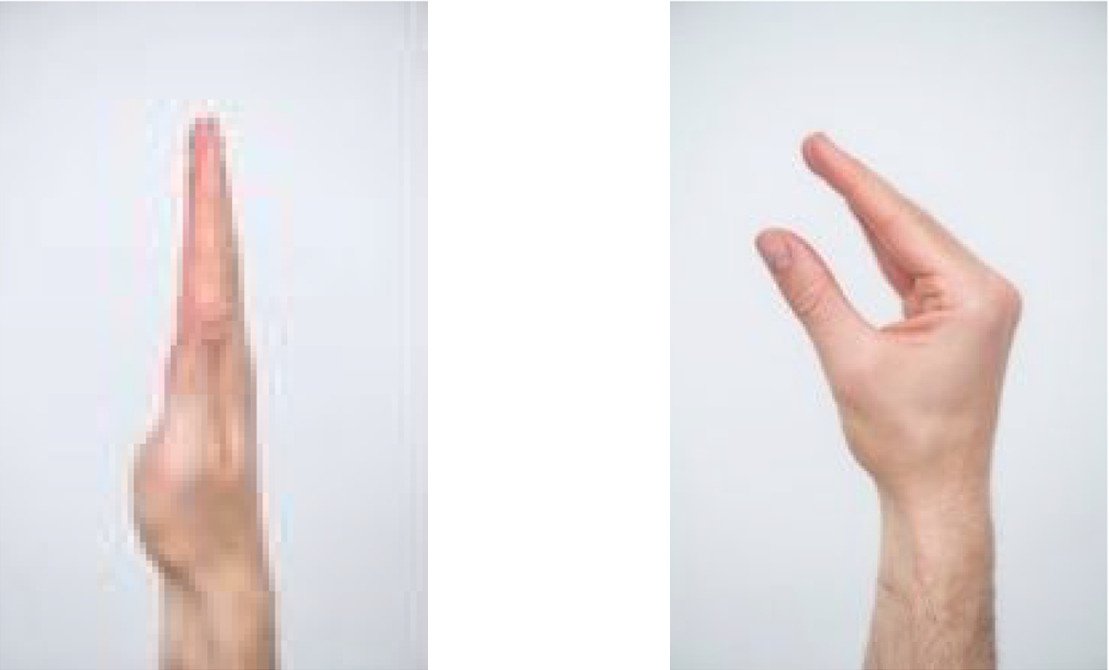
AROM DIPJ and IPJ extension then flexion (hook)	2	1 set x 10 reps, 3 times/day	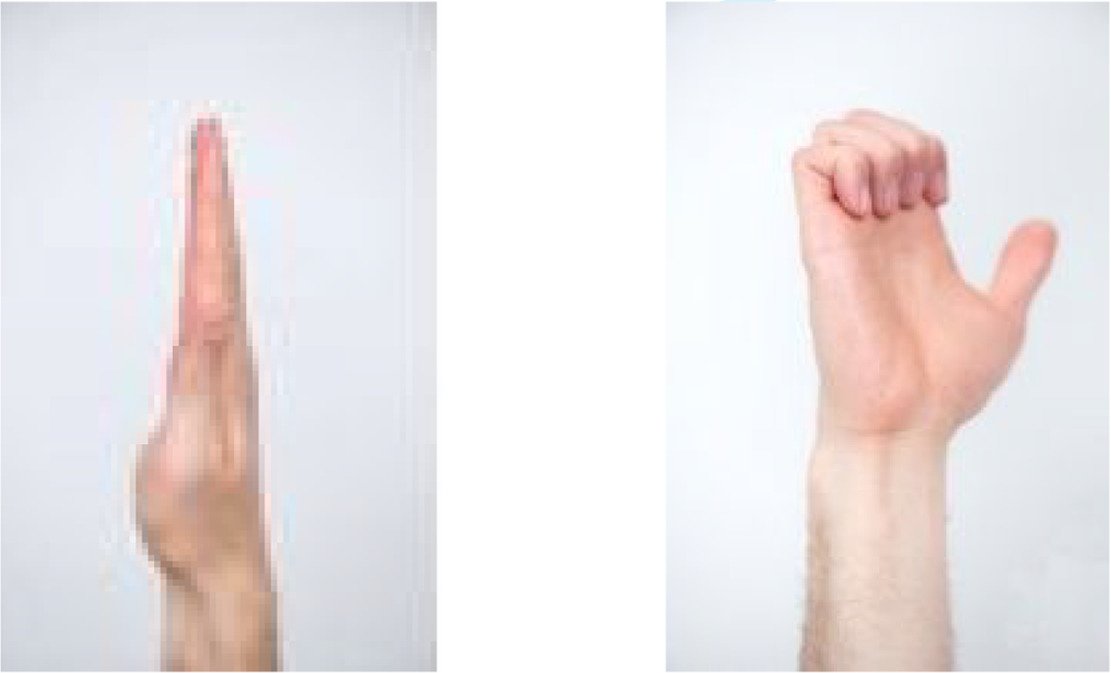
AROM full fist (fingers and thumb)	3	1 set x 10 reps, 3 times/day	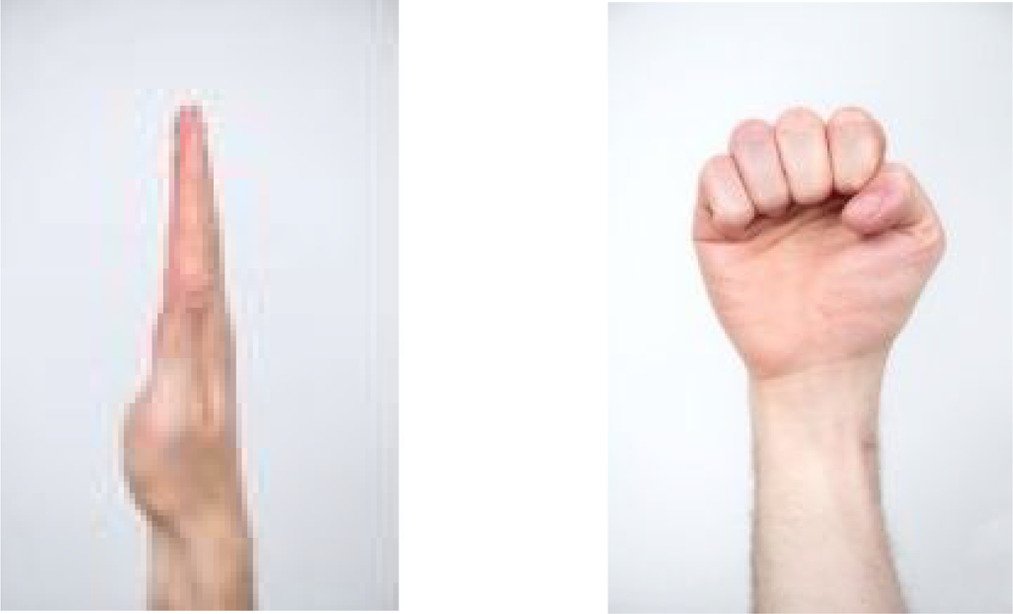
PROM full fist (fingers only)	4	3 reps x 30 s, 3 times/day	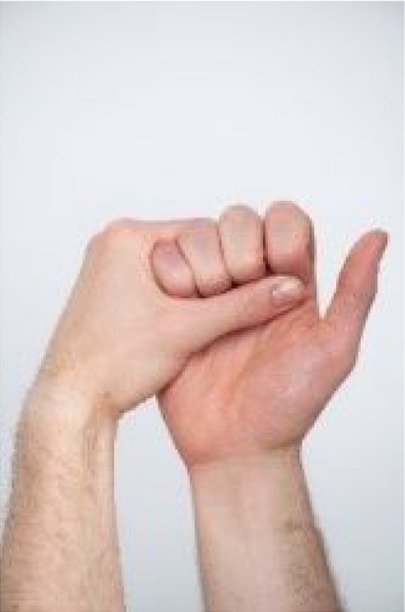
**Resistance exercises**			
Resisted shoulder extension and flexion	1	1 to 3 sets x 10 reps, 1 time/day	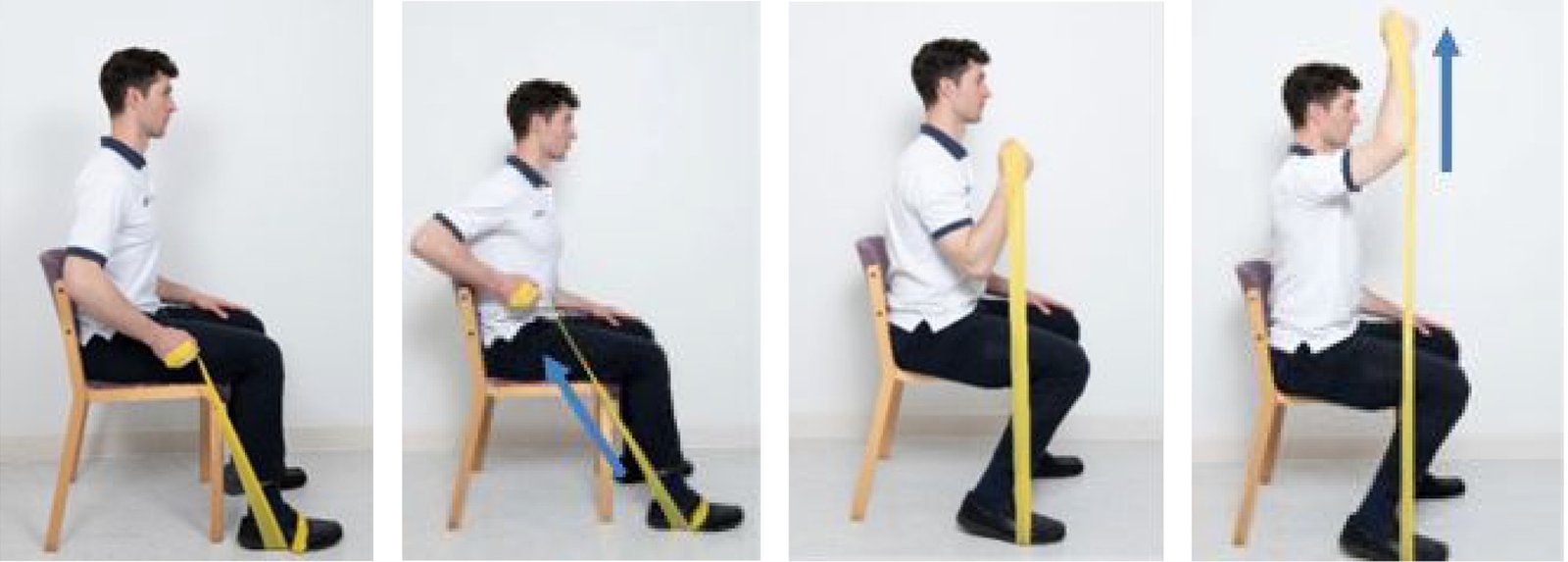
Resisted wrist extension (forearm pronated) and flexion (forearm supinated), option 1	2	1 to 3 sets x 10 reps, 1 time/day	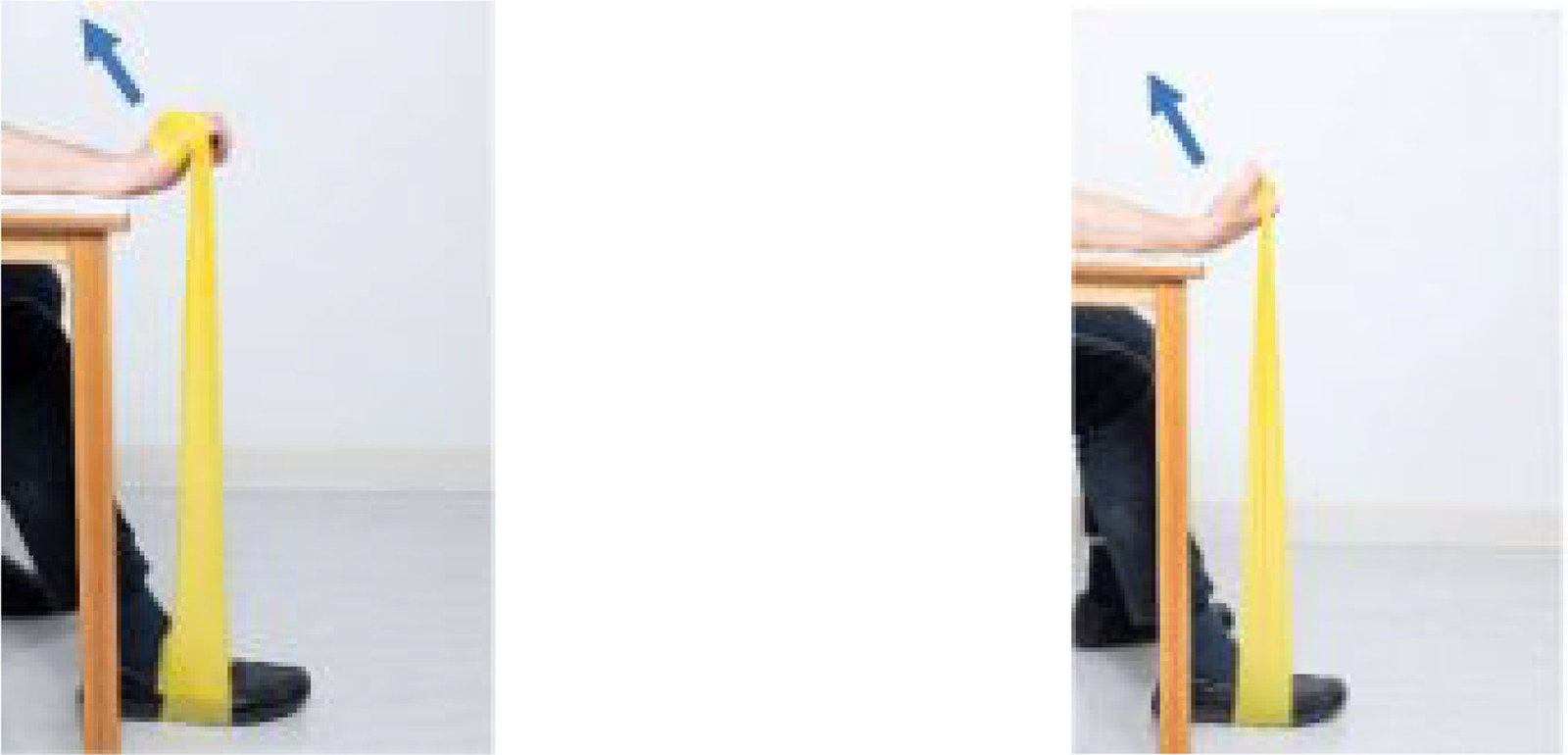
Resisted wrist extension and flexion, option 2 (neutral forearm rotation)		1 to 3 sets x 10 reps, 1 time/day	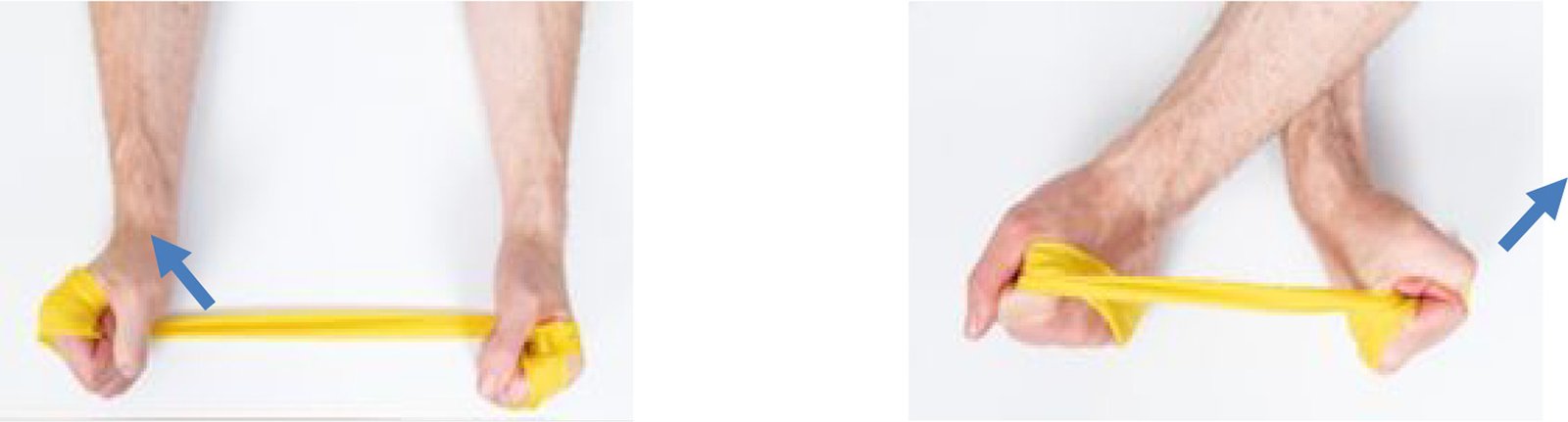
Gross grip	3	1 to 3 sets x 10 reps, 1 time/day	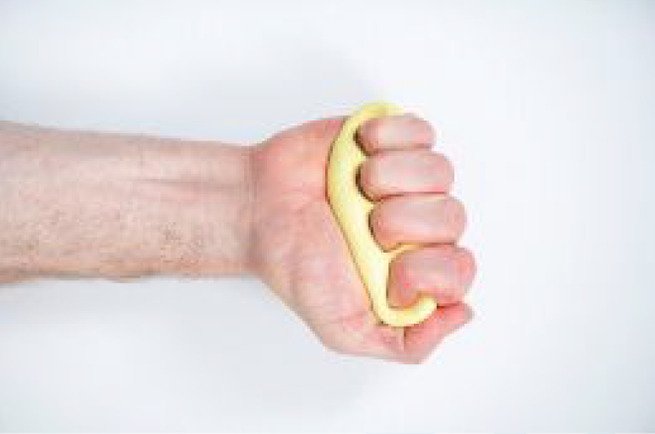
Pinch grip each finger	4	1 to 3 sets x 10 reps, 1 time/day	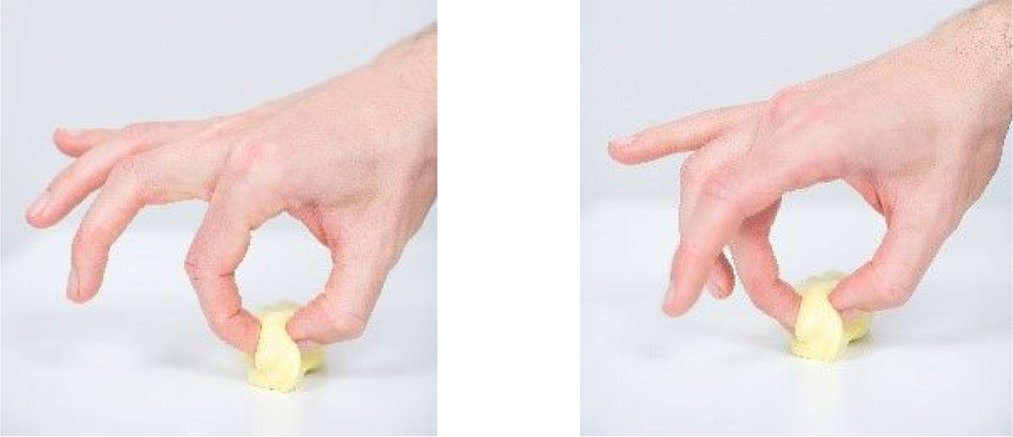

AROM, active range of motion; PROM, passive range of motion; reps, repetitions; s, seconds.

Intervention providers and training: Intervention providers were qualified NHS physiotherapists or occupational therapists in the related therapy services of recruitment sites. WISE was a pragmatic trial, so therapists did not require specific expertise or experience but must have completed training on the intervention before providing it. The trial physiotherapist (WS) provided this training face-to-face or online via video. Training lasted 90 to 120 minutes and included an overview of the trial design, a description of usual care advice, how to deliver the intervention, and how to complete trial documentation. After training, therapists were given a manual for future reference that concisely described how to deliver the intervention and complete trial documentation.

Structure and mode of delivery: Participants were offered three one-to-one sessions with a therapist. The first session was 40 to 60 minutes, face-to-face or by video, and took place immediately after usual care advice was provided or as soon as possible afterwards (aiming for within three weeks). The initial session had to be face-to-face or by video so that therapists could demonstrate the exercises then assess the participant performing them. Follow-up sessions lasted up to 30 minutes, were face-to-face or by video or telephone, and aimed to be done within 12 weeks of the first session at a schedule decided by the therapist and participant. Remote sessions were only permitted at therapy departments that normally used these. The number and duration of therapy sessions were based on what was necessary to deliver the intervention and what was considered feasible to deliver for this high-volume condition.

Participants were given the WISE paper workbook immediately following recruitment or at the first therapy session. Participants who provided a contact email address during the consent process were automatically emailed login instructions to an online version of the workbook to use if they wanted to. This online workbook mirrored the paper version, but contained advice and exercise demonstration videos, though the latter were accessible in the paper workbook via QR codes and URLs. To perform the resistance exercises, participants were also given four resistance bands (Flexil Premium Resistance Exercise Bands, UK) and four tubs of therapeutic putty (Flexil Therapy Putty, UK) which were colour coded according to their resistance level (yellow = light, red = medium, green = heavy, blue = extra heavy).

Assessment and advice: At the first session, the therapist assessed the participant as per their normal practice then briefly explained the different advice sections in the WISE workbook (Table III). The advice incorporated several elements used in the AFTER trial, adapted for the target population.^29^ This advice included the potential benefits of exercise and reassurance about the capacity to exercise, in part, to promote self-efficacy.^30^ The therapist also reminded the participant to use the online workbook if they wanted to. At review sessions, the therapist re-assessed the participant and checked their exercise performance and progress with resuming usual activities. personalized feedback was provided accordingly. In all sessions, therapists addressed any participant queries and reinforced specific advice in the workbook whenever relevant. Therapists also provided additional individualized advice if clinically indicated.

**Table III.** Overview of advice in the Wrist Injury Strengthening Exercise (WISE) workbook.

Section	Contents
Introduction	Explains that the workbook contains information that will help them with their recovery, including advice and exercises.
About your wrist	Explains the wrist’s bony anatomy and normal movements.
What is a wrist fracture?	Explains what a wrist fracture is and initial treatments i.e., surgical or non-surgical.
What can I expect after a wrist fracture?	Explains: Common symptoms after a wrist fracture Normal fracture healing (6 to 8 weeks) and recovery durations (6 to 12 months)
What can I do to help my wrist get better?	Covers: Guidance about removable splints Managing pain by using pain medication, using cold or heat, and dealing with flare-ups Managing dry skin Scar management for those treated surgically Managing swelling with elevation, exercise and massage if helpful Returning to usual activities
Returning to your usual activities	Explains the negative consequence of fear avoidance thoughts and behaviours and the positive effect of positive thoughts and increasing activity levels.
Practicing everyday activities	Guidance about practicing daily activities after removal of immobilization and how these can be progressed with examples.
Work	Advises participants who are worried about returning to work who they can contact for support e.g. their manager, their GP, Citizen’s Advice Bureau.
Driving	Information about what to consider when returning to driving with links to the DVLA and Highway Code.
Give yourself goals	Covers The benefit of setting short and long-terms goals What SMART goals are, how to set them, and gives examples Why planning where and when to do exercises is helpful and what to consider when doing this
Exercises	Covers: How exercise will help How long exercises should be done for Guidance about acceptable levels of pain during and after exercise How to do exercises, including how to set the intensity of resistance exercises and to progress these How often to do exercises
What to do if you have problems	Explains the normal duration and trajectory of recovery and who to contact if there are concerns.

DVLA, Driver and Vehicle Licensing Agency; GP, general practitioner; SMART, specific, measurable,achievable, relevant, and timely.

Exercise: The WISE programme includes eight mandatory exercises: four exercises target wrist and forearm flexibility, and four resistance exercises target muscle strength of the upper arm, wrist, and fingers. Therapists could also prescribe four optional finger flexibility exercises for participants if their assessment indicated they required them. This meant participants performed a total of eight or 12 exercises daily with flexibility exercises performed three times per day and resistance exercises once per day. This is a high volume of exercise, but most exercises are quick and convenient to perform. A similar volume of home exercise was prescribed in the SARAH programme and participants in that treatment group reported higher exercise adherence at four months than those allocated to usual care,^7^ indicating this volume of exercise is manageable.

At the first session, the therapist demonstrated each exercise and how to set the starting resistance exercise intensity. The participant performed three repetitions of each resistance exercise and rated their effort on an amended version of Borg’s 11-point scale^31^ which has been validated for measuring resistance exercise intensity.^32^ The target effort was 5 to 7 which corresponds to ‘hard’ or ‘very hard’ (scale was 0 to 10, higher is more effort). If needed, the therapist adjusted the resistance band or putty colour until the participant achieved the target intensity. This tailoring aimed to account for individual participants different physical capacities, symptoms, and stages of recovery. The therapist also explained guidance in the workbook on how to progress resistance exercises independently (presented with the effort scale in the Supplementary Material). This aimed to ensure participants’ resistance training stimulus remained sufficient to stimulate further muscle strength gains as they improved. At review sessions, therapists checked participants’ performance of the WISE exercise programme and the intensity of resistance exercises to reinforce how to progress exercises independently.

Table II shows the exercise programme along with prescription parameters which were informed by exercise prescription guidelines,^33^ our clinical experience, and survey findings. Participants were encouraged to exercise for at least 12 weeks to ensure the training duration was long enough for neurological and morphological muscular changes to occur.^34^

Strategies to promote exercise adherence: To promote participant adherence to the exercise programme, simple strategies that have appeared acceptable and implementable in our previous RCTs were used.^7,29,35^ At the first session, the therapist introduced the exercise planner (see Supplementary Material) and guidance on how to complete it. In the planner, participants wrote their specific, measurable, achievable, realistic, and timely (SMART) short and long-term goals, and where and when they would perform their exercises with a backup time if needed. The participant then signed the planner to affirm their intention to do their exercises. Then, the therapist introduced the 12 week exercise diary (see Supplementary Material) where participants recorded whether they performed their exercises at the prescribed frequency. An exercise planner was placed after every third week of the diary to prompt the participant to review their goal(s) and amend them as needed. At follow-up sessions, the therapist helped the participant to identify strategies to any barriers to exercise and reviewed and helped the participant modify their goals or exercise plan or both. The exercise planner, exercise diary, and guidance on how to complete these were in both the paper and online workbooks. Table IV describes the behaviour change techniques,^36^ used to support exercise adherence.

**Table IV.** Behaviour change techniques used in the therapist-supervised exercise intervention.

Behaviour change technique	Supervised rehabilitation
Graded tasks	The ‘practicing everyday activities’ section in the workbook gives advice about initially practicing easier activities and how to progress these. Examples of easier and harder activities are provided.
Reduce negative emotions	The workbook contains simple advice about the safety of exercise, movement, and activity and that pain does not always mean harm. The positive consequences of increased activity are explained. Sources of support if there is anxiety about returning to work are also provided.
Instruction on how to perform a behaviour	At session 1, the therapist explains to the participant how to set the intensity of resistance exercises and to progress these exercises independently. This is revisited at review sessions.
Demonstration of the behaviour	At session 1, the therapist demonstrates how to perform each exercise.
Behavioural practice	Participant performs each prescribed exercise and their therapist provides feedback.
Self-monitoring of behaviour	Participant records if they performed their stretching exercises three times per day and stretching exercises once per day in an ‘exercise diary’ in their workbook.
Goal setting (behaviour or outcome)	At session 1, the participant writes down their personalized short and long-term goals in the ‘exercise planner’ in their workbook using SMART principles. The therapist helps as needed.
Review goals (behaviour or outcome)	At review sessions, goals are reviewed with the therapist and amended if needed. A new ‘exercise planner’ is placed after every third week of the exercise diary to prompt the participant to review their goals and amend them if needed.
Action planning	The participant writes in the ‘exercise planner’ in their workbook where and when they will perform their exercises and a backup exercise time if needed. An exercise planner is placed after every third week of the exercise diary to prompt the participant to review their exercise plan and amend it if needed.
Behavioural contract	Participant signs the ‘exercise planner’ underneath the text ‘I will do my exercises each day’.
Problem solving	At review sessions, the therapist identifies if the participant has any barriers to doing their exercise and if so, helps them to develop strategies to overcome these barriers.

SMART, specific, measurable, achievable, relevant, and timely.

### Concurrent healthcare

Participants’ other healthcare continued normally. Additional therapy was available if participants needed it (e.g. if they developed complications, such as complex regional pain syndrome). Additional therapy and wrist-related healthcare were recorded (full details in protocol,^9^ and will be reported in the main results paper).

### Quality assurance

The trial physiotherapist (WS) monitored therapists’ intervention fidelity by reviewing therapist-completed case report forms which recorded treatment session details. These forms recorded if the participant attended, the session number, and delivery of key intervention components. Periodically, WS also reviewed audio recorded treatment session at study sites, and rated intervention delivery against a bespoke quality assurance checklist and provided feedback to individual therapists or sites, or both. During the feasibility phase, six recordings from two centres were reviewed, in the definitive trial phase recordings from nine sites were reviewed.

## Discussion

This paper describes the development, refinement, and delivery of the therapist-supervised WISE rehabilitation intervention evaluated in the definitive RCT. Separate reporting of intervention development and content may be particularly important in rehabilitation research, where intervention complexity and tailoring are difficult to capture fully within definitive trial reports alone. The present paper therefore aimed to provide a transparent account of the WISE intervention development process and intervention content using contemporary complex intervention and intervention reporting guidance. Such reporting is important regardless of whether the definitive trial demonstrates superiority or no evidence of effectiveness, because understanding intervention content and implementation is essential for interpreting trial outcomes and informing future research and clinical practice.

Previous systematic reviews have highlighted substantial uncertainty regarding the effectiveness of rehabilitation interventions after DRF, with many studies limited by methodological weaknesses, heterogeneity in intervention approaches, and inconsistent outcome reporting.^15^ A recent clinical practice guideline for DRF rehabilitation similarly identified several areas where recommendations were limited by conflicting or insufficient evidence and highlighted important gaps for future research.^37^ Furthermore, rehabilitation interventions are frequently insufficiently described. Bruder et al^15^ reported that fewer than 40% of upper limb fracture rehabilitation trials adequately described exercise interventions according to the TIDieR checklist,^17^ limiting replication and implementation. More recent reviews have also characterised the DRF rehabilitation literature as fragmented across methodologies and interventions.^38^ Together, these limitations reinforce the importance of transparent reporting of intervention development and intervention content alongside definitive trial evaluation.

A strength of this work was the use of an iterative intervention development process informed by current MRC and NIHR guidance for complex interventions. Compared with more pragmatic or less formally structured approaches to rehabilitation intervention development, the WISE intervention was developed using multiple complementary sources of information, including clinical guidelines, published rehabilitation research, NHS patient information resources, exercise and behavioural theory, stakeholder feedback from experienced therapists, PPI, and refinement through feasibility testing and embedded qualitative evaluation prior to definitive evaluation. This approach aimed to improve the intervention’s acceptability, feasibility, scalability, and clinical relevance, while maintaining sufficient flexibility to allow tailoring to individual participants within a pragmatic trial design. The intervention was also reported using TIDieR^17^ and CERT^18^ guidance to improve transparency, reproducibility, and future implementation.

Several limitations should also be acknowledged. The evidence synthesis undertaken during intervention development was pragmatic in nature and intended to inform intervention design rather than constitute a formal systematic review. In addition, the intervention was developed specifically within the context of the UK NHS and may therefore require adaptation for implementation in healthcare systems with different rehabilitation pathways, staffing models, or resource availability.

Wrist fractures are extremely common in older adults and result in pain and disability that can last for months. Despite this, high-quality evidence to guide rehabilitation provision for these patients after removal of their initial immobilization is lacking. The definitive WISE RCT is evaluating the effectiveness of usual care advice plus a therapist-supervised resistance and flexibility exercise intervention versus usual care advice alone in older adults with a DRF. Recruitment (n = 629 participants) is complete. The results will be submitted for publication in 2026, and will provide high-quality evidence to guide rehabilitation provision for these patients.^10^ If this intervention is shown to be clinically and cost-effective, future work will focus on supporting implementation through development of training resources and wider dissemination of patient materials for wider use.

**Take home message**

- Rehabilitation after distal radius fracture remains poorly standardized, with limited high-quality evidence to guide practice.

- The Wrist Injury Strengthening Exercise (WISE) intervention provides a reproducible, pragmatic rehabilitation model combining progressive upper-limb flexibility and resistance exercise with up to three therapist-supervised sessions and strategies to support exercise adherence.

- The programme offers a structured yet individually tailored approach that can be delivered by physiotherapists or occupational therapists and was specifically designed for scalability within routine clinical practice.

## Data Availability

All data generated or analyzed during this study are included in the published article and/or in the supplementary material.
